# The Eastern Hospital Inquiry

**Published:** 1891-08-29

**Authors:** 


### THE EASTERN HOSPITAL ENQUIRY.

### THE REPORT OF THE LOCAL GOVERNMENT BOARD.

The report upon the enquiry as to the charges made against this hospital, held by Mr. Headley and Dr. Bridges, the Board's inspectors, was submitted to the Managers of the Metropolitan Asylums Board on Saturday, the 22nd inst.

The report deals first with the complaints as to the food supplied to the patients. With regard to the bread, which, it was alleged, was dark in colour and frequently sour and stale, the inspectors were satisfied that it was good wholesome bread, although not of the best quality, and they thought that great care should be taken that this important article of diet should consist of the best white bread. It was admitted at the inquiry that margarine was supplied instead of butter. If the managers intended the patients to be supplied with it they should have had "margarine" substituted for "butter" in the diet table. The Board were aware that margarine was supplied in many of the workhouses, and that the inmates preferred it to natural butter, but they thought that patients recovering from fever should be allowed good fresh butter, the superiority of which over margarine could not be doubted. As to the fish, it seemed that fried haddock was the kind always supplied. Until recently the diet-table specified cod, brill, or sole, and it was not shown on whose authority it was altered. Dr. Collie stated that on his return to the hospital he found that haddock had been substituted, and as there were no complaints he continued it. The Board entirely disagreed with him in his approval of a dinner of fried haddock continuously for three weeks as a middle diet. Another complaint was that the dinners were often cold, but it was shown that hot-water tins had been provided for them, and that they were put down to the fire when patients had to wait any time for them. The Board, however, thought that the medical officer should, as far as possible, so time his professional visits to the wards as not to interfere with the eating of the dinners immediately on their arrival. The complaints as to the clothing were not substantiated. Most of the persons examined were thoroughly satisfied as to the linen and bedding, which were changed more often than was usual in ordinary households. The meat supply was not altogether satisfactory, and the potatoes did not appear to have been so good as they should have been, although the managers were paying £5 19s. a ton for them, as against £3 10s. a ton some time previously. Neither were the nursing arrangements so good as they ought to be in a fever hospital, and various recommendations for improving them were made for the consideration of the managers. In respect to the complaint made by four laundrymen as to the arrangements of the laundry, and as to their being unfairly treated by their application for assistance being refused, there did not appear to be any substantial ground for the complaint, but the reduction of the amount of soap and soda allowed them was a somewhat arbitrary proceeding. With regard to what took place on the leaving of nurse Halkin, the inspectors could not understand how it was that Dr. Collie, who did not suffer from deafness, did not hear what everyone else did. The whole thing was most discreditable to all concerned. As to the allegation that a child was poisoned through drinking carbolic wash, having regard to the evidence, the Board doubted extremely whether the child did drink any wash, and their own belief was that it was not poisoned at all. They, however, disagreed with the opinion of Dr. Collie that it was not a case where the death should have been reported to the coroner. A post-mortem examination was made, and in future the Board thought that post-mortems should not be allowed to be made until intimation had been sent to the nearest relative of the deceased persons. The incident of the holding of a Christmas tree in a ward in which there were twenty-three fever patients was severely commented upon, and the dancing which took place afterwards with the concurrence of Dr. Collie strongly condemned. With regard to the food generally, while the Board thought there were exaggerations in the statements of the complainants, they could not avoid the conclusion that very little care and intelligence were bestowed either on variation of the diet or in the method in which it was served, and that Dr. Collie had not acted in those matters with the discretion which might reasonably have been expected of him. On a review of the whole case, the Board regretted that they could not avoid the conclusion that, although many of the allegations of those who had complained were exaggerations of the facts, and others had been disproved, the administration of the hospital had not been satisfactory. The evidence of Sir J. Tilley, who for some time had been Chairman of the Committee of the hospital, seemed to show that the whole of the administration of the institution had been practically in the hands of Dr. Collie, the Superintendent. There had been no regular visitation by the Committee, and there had been no sub-committee authorized to visit the hospital in the interval between the Committee meetings. They regarded it as essential for the satisfactory working of the hospital that there should be direct and practical supervision by the Committee. The concluding passage of the report ran as follows: "As regards Dr. Collie, who is the chief officer of the hospital, it appears to the Board that for matters in connection with the actual working of the establishment, which had been unsatisfactory, he must to a great extent be held to be responsible. They refer specially to the unvarying nature of the diet, the arrangements as to disinfection, and the music and dancing which were kept up to a late hour, and with his express concurrence, in a fever ward. The Board cannot but bear in mind that in 1885, when he was suspended by the managers from the performance of his duties, they arrived at the conclusion, in connection with the enquiry which was then held as to the administration of the hospital, that his suspension should not be removed, and although, in deference to the representations which were made on his behalf, they subsequently assented to his reinstatement in office, they only did so on the express understanding that if in any other case cause of complaint was given, it would be impossible that he should be allowed to continue in the responsible position which he filled, and, after full consideration of all the circumstances, the Board feel that the interests of the hospital leave them no alternative but to require his resignation. With respect to the Matron the Board have much doubt whether, having regard particularly to the occurrences in connection with the leaving of Nurse Halkin, she has the judgment and discretion which are essential for a person filling the office she holds. While, however, they consider her conduct in this matter to have been most reprehensible, they are reluctant to adopt the extreme course of requiring her resignation, and if the managers concur, in that case they will consent to her continuing in her office for a period of six months on probation. The Board feel assured that the managers will give their careful consideration to the observations of the Board in regard to matters in which changes appear to be necessary. They may add that it is satisfactory to them to learn that material alterations have already been made in respect to the dietary provided for the patients."

The report was ordered to be printed and referred to the Eastern Hospital's Committee for consideration and report, and to the General Purposes Committee with power to report.

